# The roles of binding site arrangement and combinatorial targeting in microRNA repression of gene expression

**DOI:** 10.1186/gb-2007-8-8-r166

**Published:** 2007-08-14

**Authors:** Lawrence S Hon, Zemin Zhang

**Affiliations:** 1 Department of Bioinformatics, Genentech Inc., 1 DNA Way, South San Francisco, CA 94080, USA

## Abstract

**Background:**

MicroRNAs (miRNAs) are small noncoding RNAs that bind mRNA target transcripts and repress gene expression. They have been implicated in multiple diseases, such as cancer, but the mechanisms of this involvement are not well understood. Given the complexity and degree of interactions between miRNAs and target genes, understanding how miRNAs achieve their specificity is important to understanding miRNA function and identifying their role in disease.

**Results:**

Here we report factors that influence miRNA regulation by considering the effects of both single and multiple miRNAs targeting human genes. In the case of single miRNA targeting, we developed a metric that integrates miRNA and mRNA expression data to calculate how changes in miRNA expression affect target mRNA expression. Using the metric, our global analysis shows that the repression of a given miRNA on a target mRNA is modulated by 3' untranslated region length, the number of target sites, and the distance between a pair of binding sites. Additionally, we show that some miRNAs preferentially repress transcripts with longer CTG repeats, suggesting a possible role for miRNAs in repeat expansion disorders such as myotonic dystrophy. We also examine the large class of genes targeted by multiple miRNAs and show that specific types of genes are progressively more enriched as the number of targeting miRNAs increases. Expression microarray data further show that these highly targeted genes are downregulated relative to genes targeted by few miRNAs, which suggests that highly targeted genes are tightly regulated and that their dysregulation may lead to disease. In support of this idea, cancer genes are strongly enriched among highly targeted genes.

**Conclusion:**

Our data show that the rules governing miRNA targeting are complex, but that understanding the mechanisms that drive such control can uncover miRNAs' role in disease. Our study suggests that the number and arrangement of miRNA recognition sites can influence the degree and specificity of miRNA-mediated gene repression.

## Abstract 2

A genome wide analysis of factors affecting repression of mRNAs by microRNAs reveals roles for 3'UTR length, the number of target sites on the mRNA and the distance between pairs of binding sites.

## Background

MicroRNAs (miRNAs) are small noncoding RNAs that repress gene expression by binding mRNA target transcripts, causing translational repression or mRNA degradation. Currently, 475 human miRNAs have been annotated in the miRNA registry [1], with over 1,000 miRNAs predicted to exist in human [2]. They are predicted to target one-third of all genes in the genome, where each miRNA is expected to target around 200 transcripts. Given the large number of miRNAs and potential targets, miRNAs may play a key regulatory role in many biological processes.

The biogenesis of miRNAs involves a core set of proteins to convert the longer primary transcript into the mature, approximately 22 bp miRNA [3,4]. At the DNA level, miRNAs are commonly found within introns of other genes, but others exist independently, transcribed as miRNA genes. In a few cases they are clustered together in a polycistron, as in the case of mir-17-92 [5]. Upon transcription, the primary miRNA is processed by Drosha, an RNA III endonuclease, to yield an approximately 70 bp precursor miRNA [6]. The precursor miRNA is, in turn, exported from the nucleus to the cytoplasm by exportin 5 [7,8]. The enzyme Dicer then cleaves the precursor miRNA to yield a double-stranded mature product, from which one strand, the mature miRNA, is incorporated into the RNA-induced silencing complex (RISC) [9,10].

Although miRNAs are believed to regulate their targets primarily through translational inhibition, there is increasing evidence that miRNAs can also influence the abundance of target mRNAs [11]. In both mammalian and *Drosophila *systems, miRNAs have been shown to accelerate target mRNA degradation through the normal pathway of deadenylation [12-14], consequently decreasing target mRNA abundance. In fact, Lim *et al*. [15] showed that transfection of mir-1 and mir-124 into HeLa cells caused the downregulation of a significant number of genes at the transcriptional level. In another study, Krutzfeldt *et al*. [16] reported that knockdown of mir-122 using their 'antagomir' approach resulted in changes in mRNA expression for a large number of genes. The effects of miRNA-mediated mRNA degradation are moderate [12] but, nonetheless, these reports show that expression microarrays can capture the effects of miRNA repression on target genes.

Misexpression of miRNAs or improper repression of their targets can have diverse and unexpected effects. For example, a mutation in the myostatin gene (*GDF8*) in Texel sheep creates a miRNA binding site responsive to mir-1 and mir-206 that gives the sheep their meatiness [17]. In human cancer, various miRNAs are amplified or deleted [18], or otherwise have aberrant expression, suggesting that they may behave as oncogenes or tumor suppressor genes (for reviews, see [19,20]). Lastly, miRNA expression patterns for a large set of miRNAs can classify human cancers, suggesting a possible underlying connection between miRNA expression and oncogenesis [21]. Given the complexity and degree of interactions between miRNAs and target genes, understanding how miRNAs achieve their specificity is important to understanding miRNA function and identifying their role in disease.

The rules that govern miRNA target specificity are not clear, but can, in principle, be divided into several levels. At the most basic level, the specific sequence that makes up a miRNA target site determines how well the miRNA binds to the site. One often proposed rule is that a conserved 'seed' match, consisting of bases 2-9 of the miRNA, is a reliable predictor of a miRNA-target interaction, which has been supported by mutation studies that showed that those base pairs are often sufficient for binding [22]. Many miRNA target prediction algorithms have, therefore, incorporated aspects of this rule in their predictions [22-25]. However, others question whether a seed match is either necessary or sufficient for miRNA repression: a recent paper showed that perfect base pair matching does not guarantee interaction between miRNA and target gene [26], and wobble G:U base pairs are often tolerated in target sites [27,28], highlighting the complexity of miRNA-target interactions. At the intermediate level, the configuration of miRNA target sites can affect the strength of a miRNA-target interaction. For example, Doench and Sharp [29] considered the effects of altering the spacing between two CXCR4 binding sites. In addition, Sætrom *et al*. [30] recently found a range of 13-35 bp between let-7 binding sites optimizes let-7 repression. Furthermore, target prediction algorithms in general give higher scores to interactions where the target gene contains multiple binding sites [22-25]. Despite the complexity of the rules that govern miRNA target specificity, experimental validation of these algorithms show that these methods are quite accurate and sensitive (approximately 80% in one study in *Drosophila melanogaster *[31]), supporting their use in large scale analyses.

In contrast to considering miRNA target specificity at the single miRNA-target interaction or binding site levels, another level of miRNA control may involve understanding how combinations of different miRNAs may work in concert to repress a target gene. This concept was borrowed from the study of transcription regulation, where it is well known that multiple transcription factors can regulate a target gene. One clue that multiple targeting is present in miRNA regulation as well was the observation that some genes are targeted by many different miRNAs [23,25,32]. Transfection experiments [23,29] have further shown that coexpressed miRNAs can repress a gene in a concentration-dependent manner. Finally, a study in fly and worm showed that target sites for different miRNAs are often simultaneously conserved, supporting the idea of combinatorial action by miRNAs [33]. However, the extent of this phenomenon and whether miRNAs can work in concert to repress a gene need further investigation.

In this paper, we investigate the factors that affect the degree and specificity of miRNA targeting by examining the effects of both single and multiple miRNAs targeting a gene. In the case of single miRNA targeting, we explore the relationship between features of miRNA target sites and level of repression of an mRNA using a large dataset with both miRNA and mRNA expression. To do this, we developed a relative expression (RE) metric that calculates the degree of repression of a target gene as a function of changes in the expression of a miRNA. While prior systematic genome-wide efforts used expression microarrays and *in situ *hybridization to study mRNA target expression profiles [31,34-36], we incorporate both miRNA and mRNA expression data in our method, which allows us to interrogate the effects of changes in miRNA expression on target gene expression across many samples. We focus on the trends that emerge when looking at large groups of interactions, since the relationship between miRNA and mRNA in an individual interaction can be obscured by factors that regulate that mRNA's expression, such as transcriptional and splicing regulation. The metric is used to measure the effects of various binding site characteristics on miRNA repression, including 3' untranslated region (UTR) length, number of binding sites, and the distance between binding sites.

We also describe an interesting relationship between the length of CTG repeats and miRNA repression, opening the possibility that miRNAs that bind CTG repeats may be involved in CTG repeat expansion disorders such as myotonic dystrophy type 1 (DM1). CTG repeat expansion mutations in the 3' UTR are known to play an important role in several diseases, including DM1, spinocerebellar ataxia type 8, and Huntington's disease-like 2, which are all members of a class of diseases described as dominant noncoding microsatellite expansion disorders [37]. Among CTG repeat expansion disorders, DM1 is the most prevalent, affecting 1/8,000 adults, and its symptoms are multisystemic and variable, including myotonia (delayed relaxation of muscle), muscle loss, cardiac conduction defects, cataracts, insulin resistance, and mental retardation (for reviews, see [37-39]). DM1 is caused by a CTG repeat expansion mutation in the 3' UTR of the *DMPK *gene, with the most severe forms of the disease reaching thousands of repeats. Given that there are many unknowns in DM1 pathogenesis, a possible role of miRNAs in DM1 could enhance the overall understanding of the disease mechanism and thus provide new angles for therapeutic intervention.

Besides analyzing determinants of single miRNA targeting, we also examine genes that are targeted by multiple miRNAs and find that they are an unexpectedly large class of genes with strong enrichment for transcriptional regulators and nuclear factors. Expression microarray data show that these highly targeted genes are downregulated relative to genes targeted by few miRNAs, which suggests that highly targeted genes are tightly regulated and their dysregulation may lead to disease. In support of this idea, cancer genes are strongly enriched among highly targeted genes. Together, these genome-wide analyses show that the rules influencing miRNA targeting are complex, but that understanding the mechanisms that drive such control can uncover miRNAs' role in disease.

## Results

### Single miRNA targeting

We first investigated the effects of a single miRNA targeting a gene. Since we were interested in how highly expressed miRNAs could potentially repress a target gene more strongly, we exploited data from Lu *et al*. [21] and Ramaswamy *et al*. [40], containing 89 human tumor and normal samples (across 11 tissue types) for which both miRNA and mRNA expression data are available. To estimate the degree of repression (at the transcriptional level) resulting from a miRNA binding to a target transcript, we developed a RE metric, which relates changes in miRNA expression to changes in target mRNA expression (see Materials and methods for details). In summary, for a given miRNA-mRNA interaction, the RE of the interaction pair is the ratio of average mRNA expression for the one-half of samples with 'high' miRNA expression (group A), divided by the average mRNA expression for the one-half of samples with 'low' miRNA expression (group B). In interaction pairs with significant repression, the group A samples with high miRNA expression will have lower average gene expression than the group B samples with low miRNA expression, resulting in a lower RE. It is important to note that we focus on trends of RE values rather than a single absolute RE value, since the absolute RE value may be hard to interpret; a RE value of 1.0 may mean that the miRNA is not repressing the target gene, or it could also mean that the miRNA repression of the gene is counterbalanced by factors that promote activation of the gene. We used miRNA target predictions from the PicTar algorithm [23] to define miRNA-mRNA interactions. Unless otherwise specified, the following analyses use the Lu and PicTar data. Data composing the experiments can be found in the Additional data files.

**Figure 1:**
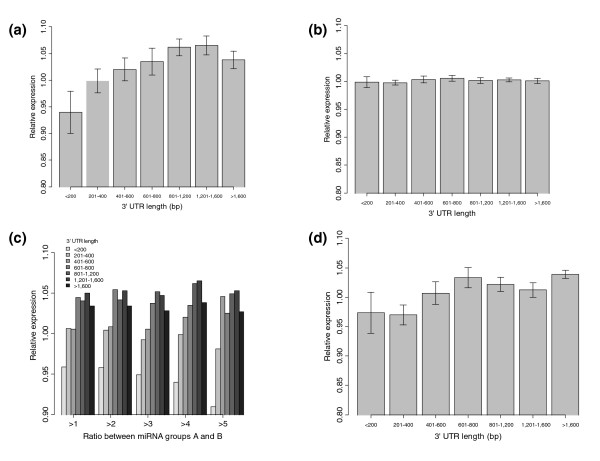
Analysis of the relationship between shorter 3' UTRs and increased repression. The error bars for observed and expected data are based on the distribution of RE values and the distribution of the permutated data, respectively. **(a) **Shorter 3' UTRs in target genes are more strongly repressed by their predicted cognate miRNAs. **(b) **The expected RE values (computed using permutation testing) show minimal deviation from 1.0, representing a lack of repression. **(c) **This trend is increasingly exaggerated when subsets of miRNAs containing larger expression ratios between groups A and B are used, especially in 3' UTRs shorter than 200 bp. **(d) **The same trend of increased repression in shorter 3' UTRs is observed using a different target prediction algorithm, rna22.

We first asked if the 3' UTR length of a gene affects miRNA repression. To counteract the effects of differing numbers of binding sites, we considered only miRNA-mRNA interactions for which the mRNA was predicted to have only one target recognition site for that miRNA (but could contain binding sites predicted to be responsive to other miRNAs; Additional data file 1 illustrates the different analyses). The relationship between 3' UTR length and degree of repression by cognate miRNAs, as measured by the RE metric, is shown in Figure 1a. MiRNA-mRNA interactions containing genes with shorter 3' UTRs tend to have lower RE values (approximately 5-10%), indicating stronger repression (*P *= 4.9 × 10^-4 ^for lengths <400 versus lengths >800).

**Figure 2:**
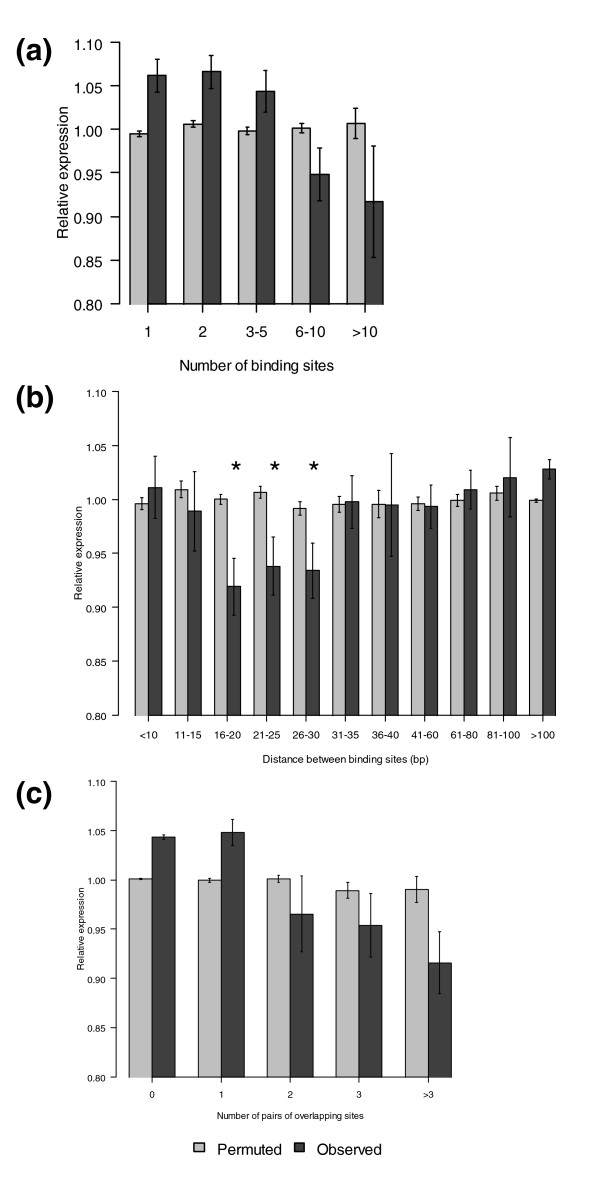
Analysis of site and gene features that affect miRNA repression. The observed values are shown in black; the expected values (computed using permutation testing) are shown in gray. The error bars for observed and expected data are based on the distribution of RE values and the distribution of the permutated data, respectively. **(a) **Target genes with more binding sites are more strongly repressed. **(b) **Pairs of binding sites targeted by the same miRNA that are between 16 and 30 bp apart (by start positions) have significantly increased repression (asterisks shown for emphasis). **(c) **Genes that have multiple pairs of extensively overlapping sites, defined to be two binding sites responsive to the same miRNA whose start positions are within 10 bp of each other, have increased repression.

To assess whether the repression observed was reasonable, we performed two analyses. In the first analysis, we calculated the expected repression for the various 3' UTR lengths if the relationship between miRNA expression and target mRNA expression were removed. By randomizing samples considered to have high or low miRNA expression, we determined the expected RE value and error at each 3' UTR length and found expected RE values of approximately 1.0 (Figure 1b), representing no repression. This showed that the changes in RE values we observed were specifically due to miRNA repression. Repeating this permutation analysis on later experiments gave similar results (Figure 2). In the second analysis, we estimated the expected magnitude of transcriptional repression for a set of predicted target genes by analyzing an independent expression data set from Lim *et al*. [15], where they transfected miRNA into HeLa cells and measured the resulting changes in expression from a panel of genes (see Materials and methods). Using PicTar predicted targets or 3' UTRs containing 7-mer seeds, the largest average downregulation for a group of predicted targets within a given transfection experiment was only 2%. If we ranked the targets by the degree of downregulation and took the subset of genes that were among the top 10% most downregulated, the maximum average downregulation for a subset for any experiment was 15%. This suggests that not many target genes are downregulated by more than 15%. Since the experiment artificially introduces a large amount of miRNA to cells, and our approach reports average changes in expression across a set of samples, a 5-10% change in expression for a group of predicted target genes represents a reasonable level of repression we might expect to see using our approach. These two analyses served to validate the use of relative expression on miRNA and mRNA expression data.

Next, we verified that the increased repression observed in shorter 3' UTRs was biologically significant and not due to artifacts in the data. First, to see if miRNAs with a larger range of expression might exhibit a larger range of target mRNA repression, we considered subsets of miRNAs that had differing ratios of expression between samples in group A versus samples in group B. As the minimum threshold of miRNA expression ratio was increased, the relative expression of genes with 3' UTRs shorter than 200 bp decreased (Figure 1c), suggesting that the RE metric benefits from greater variation in miRNA expression. Second, we considered if the result was an artifact of the miRNA target prediction algorithm used, for example, a subtle bias that would somehow preferentially identify interactions containing short 3' UTRs with low RE values. Since PicTar and other commonly used methods employ sequence conservation at the seed region as a major component of their prediction strategy, we therefore repeated the analysis using predictions from rna22 [27], a different approach that does not depend on conserved seed matches. Despite replacing the target predictions used, the same trend of greater repression found in shorter 3' UTRs was observed (*P *= 7.4 × 10^-7 ^for lengths <400 versus lengths >800; Figure 1d). Last, we tested if the result could be recapitulated using an independent data set. We obtained matching miRNA and mRNA expression data for the NCI-60 set of cell lines (see Materials and methods) and repeated the experiment using these data. Again, shorter 3' UTRs tended to be more repressed (*P *= 0.0002 for lengths <400 versus lengths >800). Thus, these results indicate that the increased repression of shorter 3' UTRs is not an artifact.

Given the confidence that we were observing a real increase in repression for shorter 3' UTRs, several explanations could account for this: first, a long 3' UTR might simply encode a complex environment in which other binding sites reside, so that the repression of the transcript by the original miRNA may be mediated by other factors; second, the probability of finding a conserved binding site increases with 3' UTR length, such that longer sequences are more likely to contain spurious sites that do not confer repression; or third, the repression could be a consequence of the physical layout of the transcript where it might be more difficult for a miRNA to find its target site within a longer 3' UTR. To further explore the final explanation, we asked if binding sites near the end of the 3' UTR might be more easily recognized by the miRNA machinery and, therefore, more likely to be repressed. We found that genes with binding sites near the end of the 3' UTR were more repressed (*P *= 0.0002 for <200 bp from the end versus >600 bp from the end, and *P *= 0.001 for <400 bp from the end versus >800 bp from the end), even when shorter 3' UTRs (<400 bp) were removed (data not shown). Since the results might be based on a combination of all three explanations, these results are consistent with the notion that 3' UTR lengths vary for functional reasons, in this case because of miRNA binding relationships.

Next we examined the effect of multiple binding sites for a given miRNA on a transcript, since for a given miRNA some genes have many more target sites than others. To reduce effects of variation in mRNA expression between tissue types due to tissue-specific effectors such as transcription and splicing factors [41], which would obfuscate the effects of miRNA repression on mRNAs, we focused only on housekeeping genes defined by Eisenberg and Levanon [42]. This resulted in a list of 155 housekeeping genes for which mRNA expression data were available, with which we plotted the number of binding sites on a target gene for a given miRNA versus RE. Figure 2a shows that genes are more repressed as the number of binding sites increases (*P *= 4.2 × 10^-6^, *n *< 5 versus *n *≥ 5). The trend remained if we used instead either NCI-60 expression data (*P *= 2.6 × 10^-7^; Additional data file 2b) or Rna22 target predictions (*P *= 0.02 for *n *≤ 2 versus *n *> 2; Additional data file 2a). This result supports previous work describing a relationship between the number of binding sites and the degree of repression [43,44]. Together, the observations that both 3' UTR length and number of binding sites affect repression show that the strength of repression is dependent on the density of binding sites within a 3' UTR.

If the number of binding sites on a target gene affects the degree of repression, the physical distance between binding sites might also affect repression efficacy. We focused on genes with 3' UTRs shorter than 800 bp since we had observed greater repression among shorter 3' UTRs, using the idea that the interactions involving shorter 3' UTRs might be more reliable. Using the remaining genes, we examined all predicted interactions for which the target gene has two or more binding sites. For each pair of binding sites on a target gene responsive to a miRNA, we computed the distance between the 5' ends of the sites, where distances less than the length of the miRNA (approximately 22 bp) represent sites that overlap. Multiple pairs of nearby binding sites responsive to the same miRNA on a given target gene were treated independently and each assigned the RE value for the interaction. We found that binding site pairs with distances between 16-30 bp were repressed by 5-10% (Figure 2b). Compared to binding site pairs with either shorter or longer distances, RE values for pairs of binding sites between 16-30 bp were significantly different (*P *= 2.2 × 10^-5 ^for *x *≤ 15 versus 16 ≤ *x *≤ 30, and *P *= 4.9 × 10^-5 ^for *x *> 30 versus 16 ≤ *x *≤ 30). It appears that binding sites with distances of 16-30 bp are in a 'sweet spot' that maximizes repression. Two sites with significant overlap might result in steric hindrance, where only one miRNA could access the two sites at a time, resulting in increased RE values. On the other hand, two sites that are farther apart might experience lower site availability due to the lower concentration of binding sites.

Additional data and recently published literature support this observation. First, similar results were observed when considering the subset of predicted interactions containing exactly two binding sites (data not shown). Second, we performed the analysis on the NCI-60 data using all genes and found increased repression for pairs of binding sites between 16 and 21 bp apart (*P *= 9 × 10^-4 ^for *x *≤ 15 versus 16 ≤ *x *≤ 21 and *P *= 2.3 × 10^-8 ^for *x *> 21 versus 16 ≤ *x *≤ 21; Additional data file 2c); though we are not certain why a smaller range of distances shows increased repression, the overlap between the results from the NCI-60 and Lu data emphasizes the repeatability of the result. Third, we verified that the result was not an artifact of binning by using a 10 bp sliding window of distances to identify regions that maximized repression. In both data sets, the most significant distances between binding sites occur between 16 and 30 bp apart (data not shown). Fourth, these results are consistent with transfection experiments in HeLa cells measuring translational repression, where it was shown that binding sites between 4 bp apart and 4 bp of overlap were more repressed than bindings sites with greater overlap, though the effect of larger distances between binding sites was not examined [29]. Additionally, Sætrom *et al*. [30] recently found maximal let-7 repression of reporter gene constructs where pairs of let-7 target sites are at distances between 13 and 35 bp, a range similar to our results. Together, these various data support the importance of the distance between sites for repression.

**Figure 3:**
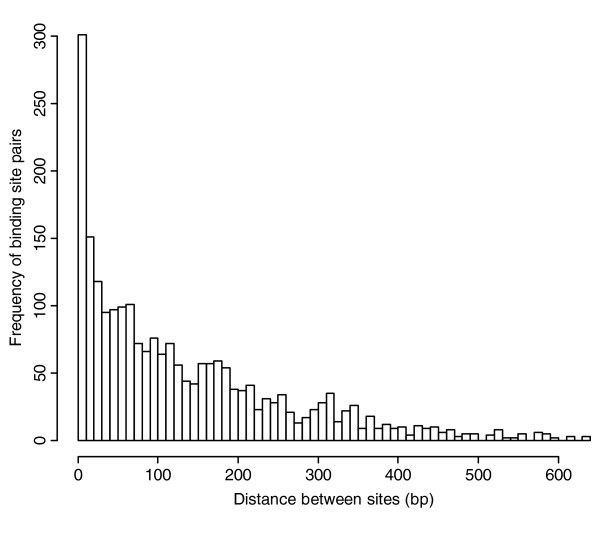
Frequency of pairs of binding sites targeted by the same miRNAs separated by a given distance. The distance between a pair of binding sites is calculated from the 5' ends of the target sites relative to the mRNA. A disproportionate number of binding site pairs are within 10 bp of each other.

While pairs of extensively overlapping binding sites were shown to have decreased repression, we also saw a disproportionate number of highly overlapping binding sites genome-wide (Figure 3). To investigate this further, we analyzed miRNA-mRNA interactions with multiple pairs of extensively overlapping sites, where a pair of extensively overlapping sites is defined to be a pair of binding sites with start positions less than 10 bp apart. Within this dataset, interactions had up to seven pairs of extensively overlapping sites. When we calculated RE as a function of number of pairs of extensively overlapping sites, we saw greater repression among genes with more pairs of extensively overlapping sites (Figure 2c; *P *= 3.8 × 10^-8 ^for *n *< 3 versus *n *≥ 3). Using NCI-60 data, interactions containing pairs of extensively overlapping sites also tended to have lower RE values compared to those that had none (*P *= 2 × 10^-5^). Therefore, although a single pair of strongly overlapping binding sites had reduced repression, this reduction can be counteracted by the presence of many binding sites.

**Figure 4:**
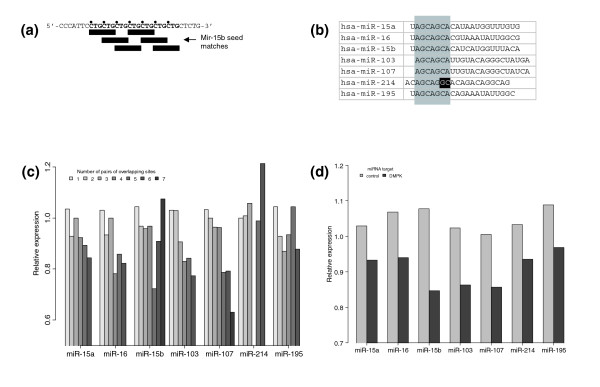
CTG repeat-binding miRNAs and their repression of pairs of extensively overlapping sites. **(a) **A diagram showing how a region of NM_173354 containing seven CTG repeats can result in six binding site seeds (CTGCTG) and five pairs of extensively overlapping sites (pairs of binding sites 3 bp apart). **(b) **Seven miRNAs containing CAG-rich seed regions that are predicted to bind to CTG repeats. Only hsa-miR-214 has mismatches in the seed region. **(c) **Number of overlapping binding sites versus relative expression for seven CTG repeat-binding miRNAs. In general, as the number of pairs of extensively overlapping sites increases, the degree of repression increases. In particular, mirs-107, -103, and -15a show a strong correlation. **(d) **Decreased relative expression of wild-type *DMPK *with respect to seven CTG repeat-binding miRNAs suggests repression of mutated *DMPK *by miRNAs could play a role in DM1. Targets with no overlapping pairs of sites served as control and showed no overall repression.

To understand how multiple pairs of extensively overlapping sites could induce greater repression, we examined individual miRNA-mRNA interactions. We found that, in many cases, a gene could embed multiple pairs of extensively overlapping sites within a small region of its 3' UTR via repetitive sequence. For example, *SNF1LK *(NM_173354) is predicted to contain six mir-15b target seed sites within a 21 bp window. Figure 4a shows how this is possible: mir-15b's seed region contains multiple CTG repeats, which would be potentially responsive to the seven CTG repeats on the 3' UTR in six different locations, creating five out of the seven total pairs of extensively overlapping sites. Given the large number of potential binding sites in a localized region of 3' UTR, the increased repression of multiple pairs of binding sites can be explained by having more binding sites available to bind to, which in turn means a greater probability of binding and thus repression. In contrast, the reduced repression of a single pair of overlapping binding sites seen in Figure 2b potentially reflects the penalty of two miRNA molecules physically blocked from binding both sites, which can be overcome by having more pairs of extensively overlapping sites.

Given that CTG repeat-containing 3' UTRs might be strongly repressed by miRNAs, we examined if CTG repeat-binding miRNAs also exhibited a correlation between number of pairs of extensively overlapping sites and repression. First we identified miRNAs with CAG repeats in their seed region besides mir-15b; these included mirs-15a, -16, -103, -107, -195, and -214 (Figure 4b). Then, for each CTG repeat-binding miRNA, RE values were calculated for target genes containing extensively overlapping pairs of sites responsive to that miRNA. Figure 4c shows that repression generally increases as the number of pairs of extensively overlapping sites increases, with the exception of mir-214, whose seed region does not contain a full complement of CAG repeats, and mir-15b, which has few targets (≤3) predicted to have six or more pairs of extensively overlapping sites. In particular, mirs-107, -103, and -15a show a strong relationship between the degree of repression and pairs of extensively overlapping sites.

Finally, we asked if CTG repeat-binding miRNAs repress wild-type *DMPK*, as a precondition to the possibility that miRNAs might be involved in the repression of mutant *DMPK *in DM1. All seven miRNAs were associated with *DMPK *repression using the RE metric (*P *= 0.02 by binomial test), with mir-107 and mir-103 repressing *DMPK *among the most at about 15% (Figure 4d). By contrast, predicted targets of the CTG repeat-binding miRNAs that contain no overlapping pairs of sites show no overall repression (Figure 4d). These data provide a preliminary validation to our postulation that miRNA repression may be involved in DM1 pathogenesis (discussed later).

### Multiple miRNA targeting

The analyses above considered the effects of single miRNAs on their target genes; we next explored the effects of multiple miRNAs targeting the same gene. Using the target predictions from PicTar [23], we identified 6,123 human genes that are predicted to be targets of one or more miRNAs. On average, these genes are targeted by 7.3 miRNAs, with some genes predicted to have as many as 65 different miRNAs targeting them. It was unlikely to observe such a large number of different miRNAs targeting a single gene by chance, since the expected number of miRNAs predicted to target a gene is approximately 2 (44,853 miRNA-mRNA interactions spread over 18,567 genes). In fact, 755 genes were targeted by more than 15 distinct miRNAs (top 50 shown in Table 1, consisting of genes targeted by ≥39 miRNAs). The enrichment for genes targeted by multiple miRNAs has been discussed elsewhere [25,32], but multiply-targeted genes as a gene class have not been fully explored.

**Table 1:** The 50 genes targeted by the most miRNAs using PicTar target predictions

Gene symbol	No. of miRNAs targeting gene	Refseq ID	Entrez gene description
ATXN1	65	NM_000332	Ataxin 1
CPEB4	63	NM_030627	Cytoplasmic polyadenylation element binding protein 4
MECP2	62	NM_004992	Methyl cpg binding protein 2 (Rett syndrome)
OTUD4	61	NM_199324	OTU domain containing 4
OGT	60	NM_003605	O-linked N-acetylglucosamine (glcnac) transferase (UDP-N-acetylglucosamine:polypeptide-N-acetylglucosaminyl transferase)
PURB	60	NM_033224	Purine-rich element binding protein B
EIF2C1	58	NM_012199	Eukaryotic translation initiation factor 2C, 1
CPEB2	54	NM_182485	Cytoplasmic polyadenylation element binding protein 2
PLAG1	53	NM_002655	Pleiomorphic adenoma gene 1
NOVA1	53	NM_006489	Neuro-oncological ventral antigen 1
DYRK1A	52	NM_101395	Dual-specificity tyrosine-(Y)-phosphorylation regulated kinase 1A
HIC2	49	NM_015094	Hypermethylated in cancer 2
RAP2C	49	NM_021183	RAP2C, member of RAS oncogene family
TRPS1	48	NM_014112	Trichorhinophalangeal syndrome I
NARG1	48	NM_057175	NMDA receptor regulated 1
NLK	47	NM_016231	Nemo like kinase
BACH2	47	NM_021813	BTB and CNC homology 1, basic leucine zipper transcription factor 2
KLF12	47	NM_007249	Kruppel-like factor 12
QKI	46	NM_206853	Quaking homolog, KH domain RNA binding (mouse)
CPEB3	46	NM_014912	Cytoplasmic polyadenylation element binding protein 3
USP6	46	NM_004505	Ubiquitin specific peptidase 6 (Tre-2 oncogene)
YTHDF3	45	NM_152758	YTH domain family, member 3
ESRRG	45	NM_206594	Estrogen-related receptor gamma
CCND2	44	NM_001759	Cyclin D2
CCNJ	44	NM_019084	Cyclin J
RSBN1	44	NM_018364	Round spermatid basic protein 1
NFAT5	44	NM_173214	Nuclear factor of activated T-cells 5, tonicity-responsive
CAMTA1	44	NM_015215	Calmodulin binding transcription activator 1
CNOT6	43	NM_015455	CCR4-NOT transcription complex, subunit 6
E2F3	43	NM_001949	E2F transcription factor 3
CHES1	43	NM_005197	Checkpoint suppressor 1
ANK2	43	NM_001148	Ankyrin 2, neuronal
MAP3K3	43	NM_002401	Mitogen-activated protein kinase kinase kinase 3
DNAJC13	42	NM_015268	Dnaj (Hsp40) homolog, subfamily C, member 13
MNT	42	NM_020310	MAX binding protein
PPARGC1A	41	NM_013261	Peroxisome proliferative activated receptor, gamma, coactivator 1, alpha
TRIM2	41	NM_015271	Tripartite motif-containing 2
ZNF238	41	NM_006352	Zinc finger protein 238
PAFAH1B1	41	NM_000430	Platelet-activating factor acetylhydrolase, isoform Ib, alpha subunit 45 kDa
HMGA2	41	NM_003483	High mobility group AT-hook 2
FNDC3B	41	NM_022763	Fibronectin type III domain containing 3B
FBXO33	40	NM_203301	F-box protein 33
STC1	40	NM_003155	Stanniocalcin 1
CPD	40	NM_001304	Carboxypeptidase D
CHD9	40	NM_025134	Chromodomain helicase DNA binding protein 9
KIAA0261	40	NM_015045	Kiaa0261
RNF38	40	NM_022781	Ring finger protein 38
BAZ2A	39	NM_013449	Bromodomain adjacent to zinc finger domain, 2A
CBFA2T3	39	NM_175931	Core-binding factor, runt domain, alpha subunit 2; translocated to, 3
FNDC3A	39	NM_014923	Fibronectin type III domain containing 3A

**Figure 5:**
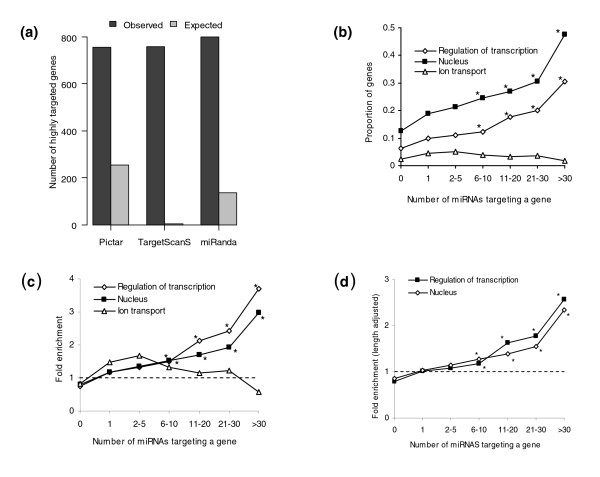
Abundance and functional enrichment of genes targeted by many distinct miRNAs. **(a) **The observed number of genes targeted by many miRNAs is dramatically greater than the expected number for all three algorithms. The threshold for the number of miRNAs to be considered highly targeted is defined to be one standard deviation more than the average number of miRNAs predicted to target a gene. **(b) **A large proportion of genes targeted by many miRNAs are transcriptional regulators and nuclear genes, but this enrichment decreases as the number of miRNAs is reduced. Genes involved in ion transporters do not show this trend. In (b-d), asterisks denote *P *< 0.01. **(c) **Enrichment, instead of proportion (as before), is shown of transcriptional regulators and nuclear genes for highly targeted genes, with the same enrichment for highly targeted genes. The expected enrichment for a random set of genes targeted by any number of miRNAs is 1.0 (that is, no enrichment), shown by the dotted line. **(d) **The enrichment of transcriptional regulators and nuclear genes among highly targeted genes remains after controlling for 3' UTR length.

To test whether the existence of so many highly targeted genes could occur by chance, we computed the expected number of genes targeted by more than 15 miRNAs using permuted data, where we scrambled the miRNA-gene relationships while keeping the number of targets per miRNA and miRNA family characteristics intact (see Materials and methods for details). On average, only 255 genes were expected to be targeted by more than 15 miRNAs (Figure 5a; *P *< 0.001). Repeating the analysis using target predictions from TargetScanS [24] and miRanda [25], similarly large differences between observed and expected number of highly targeted genes were found (Figure 5a), controlling for the possibility that the existence of highly targeted genes is due to algorithm-based biases.

The enrichment of highly targeted genes suggested that this could be a unique set of genes having common function. To test this, we performed a Gene Ontology (GO) analysis of genes targeted by more than 30 miRNAs. Table 2 shows GO categories that are the most significant in overrepresentation of these highly targeted genes. About one-third of these genes are involved in transcriptional regulation (*P *= 4 × 10^-12^), and nearly half encode nuclear proteins (*P *= 2 × 10^-15^), shown in Figure 5b. The fact that many miRNAs target the same transcriptional regulators and other nuclear genes suggests that an important means of gene regulation by miRNAs involves the direct repression of these target genes in order to trigger downstream effects. Additionally, 25% of genes are involved in developmental processes, consistent with the important role that miRNAs play in development [34,45]. While it has been previously shown that transcription regulators and development genes are commonly targeted by miRNAs [25,32], these results show that some are targeted by a disproportionate number of miRNAs, suggesting they are under particularly strong miRNA regulation. Interestingly, the enrichment for these GO categories is dependent on the number of distinct miRNAs; no such selection was observed when considering genes targeted by fewer than five miRNAs (Figure 5b,c). The strong enrichment for various gene categories and the correlation of number of miRNAs targeting a gene and category enrichment supports the notion that highly targeted genes represent a real functional class of genes.

**Table 2:** Gene Ontology categories overrepresented among the 165 genes targeted by more than 30 miRNAs

Category	Term	Count	%	*P *value
Biological Process 5	Regulation of nucleobase, nucleoside, nucleotide and nucleic acid metabolism	51	30.91	5.55E-12
Biological Process 4	Homophilic cell adhesion	15	9.09	1.82E-11
Biological Process 5	Transcription	51	30.91	1.96E-11
Biological Process 4	Regulation of cellular metabolism	54	32.73	9.26E-11
Biological Process 3	Nervous system development	23	13.94	1.49E-10
Biological Process 3	Regulation of metabolism	54	32.73	1.71E-10
Biological Process 3	Regulation of cellular physiological process	61	36.97	2.46E-09
Biological Process 3	Cell-cell adhesion	15	9.09	1.10E-08
Biological Process 4	Nucleobase, nucleoside, nucleotide and nucleic acid metabolism	56	33.94	5.15E-06
				
Cellular Component 3	Nucleus	72	43.64	1.31E-08
Cellular Component 3	Intracellular membrane-bound organelle	79	47.88	2.91E-04
				
Molecular Function 3	DNA binding	49	29.70	1.12E-08
Molecular Function 4	Transcription factor activity	30	18.18	8.45E-08
Molecular Function 3	Metal ion binding	64	38.79	1.87E-07
Molecular Function 3	Cation binding	60	36.36	6.06E-07
Molecular Function 5	Zinc ion binding	35	21.21	6.33E-05
Molecular Function 4	Calcium ion binding	23	13.94	6.74E-05
Molecular Function 4	Sequence-specific DNA binding	14	8.48	4.49E-04
Molecular Function 5	Transcription corepressor activity	6	3.64	1.12E-03
Molecular Function 3	Transcription corepressor activity	6	3.64	1.69E-03
Molecular Function 5	Calcium-transporting atpase activity	3	1.82	6.05E-03

Significant terms (*P *< 0.01) from levels 3-5 of each ontology are shown.

Next, we explored the impact of 3' UTR length on the number of miRNAs predicted to target a gene. Since genes targeted by multiple miRNAs necessarily have more miRNA binding sites, it was possible that highly targeted genes result solely from having longer 3' UTRs. Therefore, the enrichment for transcriptional regulators among highly targeted genes, for instance, might result from transcriptional regulators in general having longer 3' UTRs. To control for this possibility, we performed a permutation-based experiment to see if random genes having the same average 3' UTR length and gene set size as the test category would be equally enriched for genes targeted by multiple miRNAs (see Materials and methods for details). We found that for both transcriptional regulators and nuclear factors, the enrichment of genes targeted by 10 or more miRNAs is still statistically significant after controlling for 3' UTR length (Figure 5d). Thus, highly targeted genes are enriched for transcriptional regulators and nuclear factors independent of 3' UTR length.

**Figure 6:**
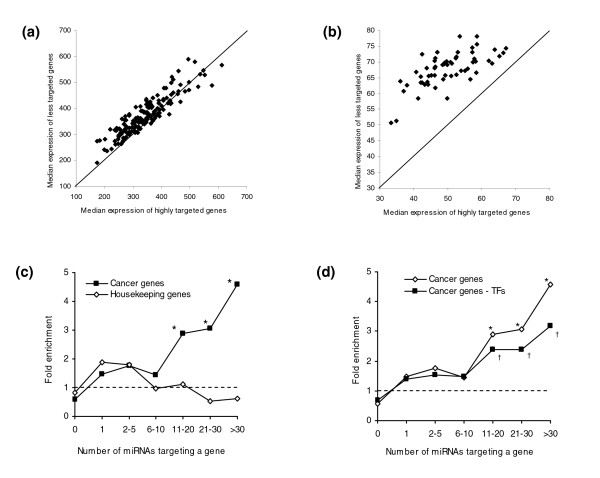
Downregulated expression and enrichment of cancer genes among highly targeted genes. **(a) **In a comparison of highly targeted genes (n > 20) versus less targeted genes (1 ≤ n ≤ 5) in normal tissue samples [46], 121 out of 158 samples exhibited decreased expression among highly targeted genes (*P *= 1 × 10^-11^). **(b) **Out of 58 NCI60 cancer cell line samples, 58 exhibited decreased expression among highly targeted genes (*P *= 7 × 10^-18^). **(c) **Highly targeted genes are enriched for cancer genes, with cancer genes targeted by >30 miRNAs having the most enrichment. In (c,d), asterisks denote *P *< 0.01 and crosses denote *P *< 0.05.**(d) **This enrichment for cancer genes remains after removing transcriptional regulators, which are prevalent among cancer genes and, as shown earlier, overrepresented among highly targeted genes.

We next examined if highly targeted genes might be more tightly regulated, since more miRNAs could potentially repress them at any given time. Given this hypothesis, highly targeted genes might be expected to have, on average, lower expression than less targeted genes. When analyzing expression microarray data from a panel of normal tissues [46], we found that, in a majority of samples, highly targeted genes (*n *> 20) in fact exhibited a lower median absolute expression than less targeted genes (1 ≤ *n *≤ 5; *P *= 1 × 10^-11^; Figure 6a). More strikingly, in available NCI60 cancer cell line data all 58 samples had lower expression among highly targeted genes (*P *= 7 × 10^-18^; Figure 6b). These results support a combinatorial model of miRNA regulation, where different miRNAs simultaneously repress highly targeted genes to yield a lower average expression.

The possibility that many of these genes are tightly guarded by multiple miRNAs suggested that the dysregulation of these genes could lead to undesirable events, such as the development of diseases like cancer. Considering cancer-related genes from the Cancer Gene Census [47], we found that the enrichment for cancer genes was most pronounced in genes targeted by >30 miRNAs (over four-fold enrichment, *P *= 2 × 10^-4^; Figure 6c). By contrast, housekeeping genes, which are highly conserved, had no enrichment, removing the possibility that conserved genes in general have more miRNAs targeting them (Figure 6c). We tested whether the enrichment for cancer genes was simply due to the overrepresentation of transcription factors, which are known to be common among cancer genes, but the enrichment remained after subtracting out transcription factors (Figure 6d).

To determine whether cancer genes as a class are preferentially targeted by multiple miRNAs, we computed the average number of miRNAs targeting cancer-related genes. On average, 5.6 miRNAs targeted the cancer genes, over 7 standard deviations higher than what would be expected by chance (*P *= 2 × 10^-13^). The same increased multiple targeting of cancer genes was observed when using two other algorithms, TargetScanS and miRanda [24,25] (*P *= 4 × 10^-18 ^and *P *= 4 × 10^-8^, respectively). Likewise, pruning miRNA families with multiple members also did not attenuate the signal (data not shown). Although many of the predicted miRNA targets are not experimentally verified, the overwhelming trends we observed will likely hold despite potential noise in the datasets.

## Discussion

In this study, we examined both single and multiple targeting of miRNAs and their effects on repression. Because of the far-ranging effects of miRNA repression, it is likely that miRNAs are involved in many diseases as well. In the case of multiple targeting, we show that cancer genes tend to be targeted by more miRNAs, supporting the notion that miRNAs play a role in cancer. In the case of single targeting, we describe below a possible relationship between miRNAs and DM1, using observations about the repression of genes containing multiple pairs of overlapping binding sites. These links to diseases underline the importance of studying the mechanisms behind miRNA targeting, which we discuss in the following.

### Understanding multiple targeting

The fundamental motivation for having multiple miRNAs target a gene is so that these presumably important genes can be regulated in a variety of conditions such as different tissue types or transcriptional programs. While it is known that some genes have recognition sites for multiple different miRNAs, it is uncertain whether multiple miRNAs simply supplement each other in different conditions or they act in concert to provide enhanced gene repression. In a simple model, each miRNA would be independently responsible for regulating genes that need to be repressed for a given condition (for example, a specific tissue type). For genes that need to be active under a number of specific conditions, a different miRNA could be expressed under each condition that that gene needed to be regulated. MiRNAs would, therefore, act independently of each other, so that in the case that multiple miRNAs happened to be expressed simultaneously, there would not necessarily be any enhanced repression.

A more intriguing model involves multiple miRNAs working in concert to repress a gene. In this case, two different miRNAs expressed independently could each repress a given gene. If both miRNAs are expressed simultaneously, however, then that gene is much more strongly repressed than the repression exerted by each miRNA on its own. This coordinated regulation is achieved in transcriptional regulation when two transcriptional factors interact in a transcriptional complex while binding to the promoter of a gene. Since miRNAs are much smaller than transcription factors and, therefore, have little of a binding interface, it is unlikely that miRNAs could directly interact. It is possible that miRNA complexes (as part of a RISC complex) could instead interact, but the binding interfaces to these complexes would need to exhibit some unique characteristic of that miRNA to differentiate one complex from another. Another possibility is that two binding sites responsive to different miRNAs may have different repressive potential depending on the distances between the two sites, similar to the results shown above for a single miRNA on multiple sites. Independent of the mechanism, the fine degree of regulation allowed by coordinate miRNA repression makes this an appealing model that deserves further attention. Our observation that genes targeted by more miRNAs tend to be repressed more than genes targeted by fewer miRNAs is consistent with the combinatorial model where both the degree and specificity of miRNA regulation can be modulated by various combinations of relevant miRNAs.

### CTG repeat-binding miRNAs and link to myotonic dystrophy

Our observation that CTG repeat length correlates with miRNA repression led us to surmise a possible role for this phenomenon in disease, in particular DM1. If a 3' UTR were to gain CTG repeats, it would be possible to abnormally repress that transcript, affect the stoichiometry of free to bound CTG-repeat binding miRNAs, or otherwise disrupt CTG-repeat binding miRNA function. We focused on DM1 because CTG repeat expansion in *DMPK *has been shown to be the cause of the disease and because the detailed mechanism for DM1 pathogenesis remains unresolved.

We therefore propose that miRNA repression of CTG repeats plays a role in the mechanism of DM1. In this model, CTG repeat-binding miRNAs, such as mir-107 and mir-103, preferentially bind to the mutated *DMPK *transcript. This could have two miRNA-leaching effects: first, the amount of unbound miRNA that would normally be regulating other genes is reduced and could no longer repress other target genes; or second, the strength of the repression due to long CTG repeats could result in the sequestration of large amounts of miRNA machinery and prevent normal miRNA repression in general. MiRNA involvement could have significant consequences on known proteins in DM1 disease progression. In the current view, the CTG repeat expansion triggers sequestration of the *DMPK *transcript into nuclear foci [48] along with *MBNL *[49], implicating *MBNL *as a key player in DM pathogenesis. Instead of the prevalent view that *MBNL *binds directly to DMPK mRNA, *MBNL *might instead be responsive to the complex with miRNAs binding to the *DMPK *3' UTR.

This proposed relationship between *MBNL *and miRNAs might explain why colocalization of *MBNL *with RNA foci does not necessarily trigger DM1 downstream events [50] and why MBNL1 apparently binds to other types of repeats even better than CTG repeats [51]; in both cases, miRNA-binding to CTG repeats might mediate this interaction. One potential complication to this theory is that while, traditionally, miRNA biogenesis assumes that mature miRNA is active only within the cytoplasm, miRNA binding to *DMPK *transcripts would require that miRNAs and their machinery exist within the nucleus. In fact, recent evidence has shown that miRNAs are also active within the nucleus [52], facilitated by a nuclear import mechanism [53].

Several lines of evidence support our theory that miRNAs might be involved in DM1. First, as we showed earlier, miRNA repression increased as the length of CTG repeats increased, suggesting a relationship between CTG repeat length and miRNA repression. Second, we also showed that wild-type *DMPK *is responsive to repression by CTG repeat-binding miRNAs. Additionally, disrupting miRNA biogenesis through the knockdown of Dicer has been shown to increase *DMPK *expression [54], suggesting that miRNAs regulate *DMPK*. Finally, the model implies that CTG repeat-binding miRNAs should be expressed in the tissues that exhibit DM1 symptoms. Using published mouse miRNA expression data [55], we found that our strongest candidates, mir-107 and mir-103, are indeed strongly expressed in brain, heart, and muscle. Together, these results support a role for miRNAs in DM1 pathogenesis, and, in particular, highlight mir-107 and mir-103 as attractive candidates for binding to *DMPK*.

### Observations about the relative expression metric

The RE metric as used in this paper is unique compared to previous efforts in understanding miRNA targeting in that both miRNA and mRNA expression data have been used together to measure miRNA repression. Previous efforts utilizing only expression microarray or *in situ *hybridization data indirectly measured differential expression of mRNA targets by taking advantage of knowledge about tissue- or stage-specific expression of miRNAs. For example, since mir-1 tends to be expressed in skeletal muscle, it is expected that targets of mir-1 should be downregulated in muscle samples. However, miRNA expression characteristics can be inferred for only a limited number of miRNAs for a given sample type. In this approach, the experimentally derived expression of many different miRNAs is known for multiple samples, making it possible to calculate the differential expression of target genes using measurements of actual miRNA expression levels. Using this approach, we performed an *in silico *genome-wide assessment of binding site-specific characteristics of miRNA repression, including the number of binding sites, distances between binding sites, pairs of extensively overlapping sites, and length of the 3' UTR. While some of these morphological features have been previously discussed as factors linked to or contributing to miRNA repression, they had generally been studied under specific and limited experimental conditions or in the context of miRNA target predictions without regard to expression data.

Because the Lu *et al*. dataset [21] used here comprises a heterogeneous set of samples drawn from different tissue types and cancer status, tissue- and cancer-specific gene expression complicates analysis of miRNA repression. For this reason, housekeeping genes, which are universally expressed, served to reduce variation in gene expression across different tissues due to non-miRNA specific effects. It is also for this reason that we chose to employ the RE metric and not a correlation metric. Because of the complexity of the data, the expected anti-correlations that correspond to repression tend to be very slight and, therefore, difficult to interpret. Additionally, the RE metric corresponds more closely to the concept of degree of repression, where smaller RE values correspond to down-regulation and thus greater repression.

While larger changes have been observed in translational inhibition by miRNAs compared with transcriptional repression, the relatively small changes in RE values that we observed nevertheless emphasize the importance of miRNA-mediated transcriptional repression. As we showed, the 5-10% lower RE values for a set of gene interactions is in line with the average repression of target genes in cells transfected with miRNA in Lim *et al*. [15]. Importantly, both calculations are based on a large number of gene targets, and, therefore, subject to various sources of noise and uncertainty. These include the possibility that some genes might be more strongly repressed by a miRNA than others, that some gene targets might have been mis-predicted by PicTar, or that some gene targets might only be expressed or responsive to a miRNA in certain tissues. Despite these potential sources of noise, our ability to detect the observed trends shows that the results are applicable genome-wide and emphasize the role of miRNA repression at the transcriptional level. Since it appears that the same sequence features (that is, the distance between binding sites) can influence repression both at the translational level [29] and transcriptional level (shown here), this suggests that the mechanisms driving miRNA-mediated transcriptional and translational repression may be linked.

One unexpected observation was the presence of RE values greater than 1.0 in various analyses. This effect is possible if considered within the context of total gene regulation, where multiple factors compete to up- and downregulate a gene. In this scenario, transcription factors and miRNAs that are simultaneously expressed exert opposing effects on the regulation of genes. If, on balance, a gene experiences greater transcriptional activation than miRNA repression, then this gene could exhibit RE values greater than 1.0 despite the miRNA repression. This apparent upregulation in the presence of miRNA repression should not be considered surprising given the belief that miRNAs serve to fine-tune gene regulation in feedback loops, increasing the precision and robustness of gene expression [31,56,57].

We anticipate that the RE metric will be able to reveal additional features of miRNA repression when applied to larger datasets containing more uniform data, such as those containing the same tissues or cancer state. Some potential experiments include testing for cooperative effects of multiple miRNAs working together to repress a gene, interactions between miRNAs and transcription factors when targeting a gene, and binding site specific effects, such as the importance of the seed region or the tolerability of G/U mismatches.

## Conclusion

Through the integration of miRNA target predictions and miRNA and mRNA expression data, we have been able to analyze features of single and multiple targeting. We first showed that a relative expression metric could be used to measure the degree of repression by miRNAs, demonstrating that 3' UTR length, number of binding sites responsive to a miRNA, and distance between two binding sites responsive to the same miRNA are all important factors that influence miRNA repression. Interestingly, we also showed that miRNA repression increases as the number of pairs of extensively overlapping sites increases, in many cases due to regions of CTG repeats. This creates the possibility that miRNA repression of CTG repeats might be involved in diseases that involve expansions in CTG repeats, such as DM1, for which we had some preliminary evidence. We then analyzed genes that are targeted by multiple miRNAs and found an unexpected abundance of such genes, with significant enrichment for transcriptional regulators and nuclear genes. Exploring the notion that highly targeted genes might be more tightly regulated, we demonstrated that highly targeted genes are downregulated relative to less targeted genes. Finally, supporting the idea that dysregulation of tightly regulated genes could lead to disease, we showed that highly targeted genes were enriched for cancer genes. The results presented here show that approaches that integrate multiple types of data are a powerful way to elucidate miRNA mechanisms in both single and multiple targeting and can further our understanding of disease.

## Materials and methods

### Datasets

We utilized expression data from 89 samples from Lu *et al*. [21] and Ramaswamy *et al*. [40], for which both miRNA and mRNA expression data are available. Both datasets underwent data preprocessing as described by Lu *et al*. This resulted in 195 miRNAs and 14,546 mRNAs available to be used for analysis. We also obtained expression data from the NCI-60 set of cell lines, using miRNA expression data from Blower *et al*. [58] and mRNA data from Genelogic [59]. For the NCI-60 miRNA data, we followed the data normalization as described by Blower *et al*. and for each miRNA used the probe that had the higher median expression. For the NCI-60 mRNA data, we set a minimum log2 expression value of 5. For the multiple targeting analysis, we used, in addition, the NCI-60 cancer cell line gene expression dataset, generated by Novartis based on the Affymetrix U95v2 array platform, and the normal human tissue expression dataset based on the Affymetrix U133A array platform [46].

For the multiple targeting analysis, we used miRNA target prediction data obtained from PicTar [23], TargetScanS [24], and miRanda [25]. For PicTar, we chose the results based on conservation in human, chimp, mouse, rat, and dog. For TargetScanS predictions we chose the four species comparison and only SeedM matching to increase the number of predictions available. In the single targeting analysis, to increase the number of binding site predictions available, we used the updated PicTar algorithm [60], which includes sites that may not be fully conserved. We also used publicly available rna22 target predictions [27,61], which predicts relatively fewer sites per target gene and, therefore, was used for analyses that depend less on having multiple sites per target gene.

For the locations of predicted miRNA binding sites within the 3' UTR, we mapped the sequences onto Release 16 of RefSeq [62] transcripts. 3' UTR lengths of genes were also extracted from the RefSeq data. We obtained 340 cancer-related genes from the Cancer Gene Census [47], and 556 genes designated as housekeeping genes from Eisenberg and Levanon [42].

### Relative expression metric

The RE metric as applied to miRNA and mRNA expression data is an estimate of the degree of repression of a gene (at the transcriptional level) resulting from a miRNA binding to a target transcript. To calculate the RE for a given gene repressed by a given miRNA, for *n *samples for which both miRNA and mRNA expression data are available, we first sort the samples by miRNA expression. We then equally divide the samples into two groups, group A with low miRNA expression (samples 1 to *n*/2), and group B with high miRNA expression (samples *n*/2 + 1 to *n*). The RE is thus the ratio of the median mRNA expression of samples in group B divided by the median mRNA expression of samples in group A. Interactions with strong miRNA repression yield smaller RE values, since group B should have lower mRNA expression because of the higher miRNA expression, and group A should have higher mRNA expression because of the lower mRNA expression. The experiments are illustrated in Additional data file 1.

To focus on miRNA-mRNA interactions with the most potential signal, we considered the subset of interactions with sufficient variation among the samples. For analysis of 3' UTR lengths, we considered only miRNAs where, for each miRNA, the median miRNA expression in group B (samples with high miRNA expression) was at least four times greater than the median miRNA expression in group A (samples with low miRNA expression). In the experiment considering the number of binding sites versus repression, we only required that the two groups have different median miRNA expression to maximize the number of interactions available. Additionally, for all experiments, we removed interactions for which there was no difference in median mRNA expression between the two groups. The average relative expression for a particular condition was defined to be the median of the remaining RE values that satisfy that condition. The significance of a group of RE values was assessed using the double-sided *t*-test; for the tests shown, varying the thresholds did not significantly affect the *P *value.

To determine the expected RE values for a given experiment, we created a null distribution of experiments, where for a given miRNA randomized miRNA expression values were used so that the samples used in groups A and B would be scrambled. The expected value consists of ten permutations of this data, from which the error bar is derived.

### Maximum expected repression

To determine if a 10% repression was reasonable using the RE metric, we examined the downregulation of miRNA target genes using data from Lim *et al*. [15]. To choose a subset of miRNA predicted targets with the largest possible repression, we sorted the gene targets by the degree of downregulation and identified the top 10% most downregulated targets. A separate set of predicted targets was defined using 3' UTRs that contain 7-mer seed matches to the miRNA (positions 2-8).

### Distance between binding sites

We defined a pair of binding sites to be two binding sites responsive to the same miRNA, for which there are no other binding sites responsive to that miRNA in between them. The distance between the pair of binding sites was calculated based on the 5' end of the binding site relative to the mRNA transcript. To identify distance ranges having significantly increased repression, we used a 10 bp sliding window of distances for distances between 1 and 100 bp apart, and performed a double-sided *t*-test between RE values contained in the window versus RE values outside of the window, using a Bonferroni correction of 90 hypotheses.

### Number of miRNAs that target a gene

For each transcript, we tallied the number of different miRNAs predicted to target that transcript. For genes with multiple transcripts, a representative transcript was chosen, so that results could be reported at the gene level.

### Enrichment analysis

We used DAVID [63] to determine enrichment of types of genes within the highly miRNA-targeted genes, focusing on gene sets obtained from GO [64]. Significance was assessed using the EASE score from DAVID [63], a modified Fisher's Exact Test. Fisher's Exact Test was used to determine the significance of the enrichment of a subset of genes targeted by a range of miRNAs shown in the figures. Enrichment was calculated in the following way: if, for a gene set *R*, among all genes genome wide *G*, we observe *R*_*s *_(a subset of *R*) within *G*_*s *_(a subset of *G*), then the enrichment of *R *within this subset is $\frac{R_{s}/G_{s}}{R/G}$, where the observed proportion of *R*_*s *_is *R*_*s*_/*G*_*s *_and the expected proportion of *R*_*s *_is *R*/*G*. A multiple testing significance threshold can be conservatively defined in this enrichment analysis by applying a Bonferroni correction to yield *P *< 5 × 10^-5 ^as the adjusted *P *value required for significance (given approximately 1,000 GO categories used by DAVID).

### Statistical tests

The statistical significance of seeing a number of genes targeted by at least a lower threshold *n *miRNAs was computed using permutation testing. For a given algorithm's target predictions, we determined the number of targets a given miRNA targeted, and assigned the same number of random genes to that miRNA. In the case that a miRNA belonged to a family of miRNAs, we assigned the same set of random genes to all of the miRNAs in that family. This procedure was repeated 1,000 times, and the *P-*value could be assessed by determining the fraction of permutations that the number of genes targeted by at least *n *miRNAs was greater than the observed number of genes. To account for differences in the algorithms, for each algorithm we defined *n *to be one standard deviation more than the average number of miRNAs predicted to target a gene.

Permutation testing was also used to control for the possibility that the enrichment for transcription and nuclear factors targeted by multiple miRNAs was due to longer 3' UTRs. To do this, we created a null distribution containing 10,000 random sets of genes with the same average 3' UTR lengths and gene set sizes as those of transcription factors or nuclear proteins, respectively. From this distribution, we determined, for example, the likelihood of seeing 36 transcription factors targeted by >30 miRNAs, controlled for both 3' UTR length and gene set size, based on the number of times more than 36 genes were found to be targeted by >30 miRNAs.

To determine the significance of the number of miRNAs targeting a particular gene set, we examined the mean number of miRNAs targeting the genes in the gene set (referred to hereafter as 'score') and compared it against the null distribution. The null distribution was generated by creating 1,000 random gene sets containing the same number of genes as the test set, and calculating the score for these random gene sets. Because the null distribution is normally distributed, we could compute the *P *value based on the z-value, where Z = (observed score - expected score)/standard deviation.

### Testing for miRNA family effects

MiRNAs are known to cluster into families having highly similar binding sites. To avoid having an *a priori *definition of which miRNAs belong to a family, we instead considered the miRNAs in the context of each target gene. For a given target gene, we tracked the predicted locations of target sites for each miRNA, and we discarded the miRNAs that were predicted to bind to the same location recognized by another miRNA. This would yield a set of miRNAs with unique target sites, predicted to target a gene. This number by definition is less than or equal to the total number of miRNAs that are predicted to target a gene.

## Additional data files

The following additional data are available with the online version of this paper. Additional data file 1 illustrates the different analyses performed using the relative expression metric. Additional data file 2 contains figures using alternative data sets that support the observed trends. Additional data file 3 contains a description of the tables of the raw relative expression data and associated data found in Additional data files 4, 5, 6, 7, 8. Additional data file 4 contains relative expression values for all PicTar predicted interactions using the Lu/Ramaswamy set of expression data. Additional data file 5 contains relative expression values for all PicTar predicted interactions using the NCI-60 set of expression data. Additional data file 6 contains relative expression values for all rna22 predicted interactions using the Lu/Ramaswamy set of expression data. Additional data file 7 contains relative expression values for specific pairs of binding sites (<1000 bp apart) responsive to a miRNA, using Lu/Ramaswamy data and PicTar predictions. Additional data file 8 contains relative expression values for specific pairs of binding sites (<1000 bp apart) responsive to a miRNA, using NCI-60 data and PicTar predictions.

## Abbreviations

DM1 = myotonic dystrophy type 1; GO = Gene Ontology; miRNA = microRNA; RE = relative expression; RISC = RNA-induced silencing complex; UTR = untranslated region.

## Supplementary Material

Additional data file 1 Different analyses performed using the relative expression metric. Click here for file

Additional data file 2 Figures using alternative data sets that support the observed trends. Click here for file

Additional data file 3 Description of the tables of the raw relative expression data and associated data found in Additional data files 4-8. Click here for file

Additional data file 4 Relative expression values for all PicTar predicted interactions using the Lu/Ramaswamy set of expression data. Click here for file

Additional data file 5 Relative expression values for all PicTar predicted interactions using the NCI-60 set of expression data. Click here for file

Additional data file 6 Relative expression values for all rna22 predicted interactions using the Lu/Ramaswamy set of expression data. Click here for file

Additional data file 7 Relative expression values for specific pairs of binding sites (<1000 bp apart) responsive to a miRNA, using Lu/Ramaswamy data and PicTar predictions. Click here for file

Additional data file 8 Relative expression values for specific pairs of binding sites (<1000 bp apart) responsive to a miRNA, using NCI-60 data and PicTar predictions. Click here for file
