# Supplementary material for: The roles of binding site arrangement and combinatorial targeting in microRNA repression of gene expression
Source: Genome Biol. 2007 Aug 14;8(8):R166. doi: 10.1186/gb-2007-8-8-r166 (PMC2374997; doi:10.1186/gb-2007-8-8-r166)
Supplement: Additional data file 1 — Different analyses performed using the relative expression metric. [file gb-2007-8-8-r166-S1.pdf]

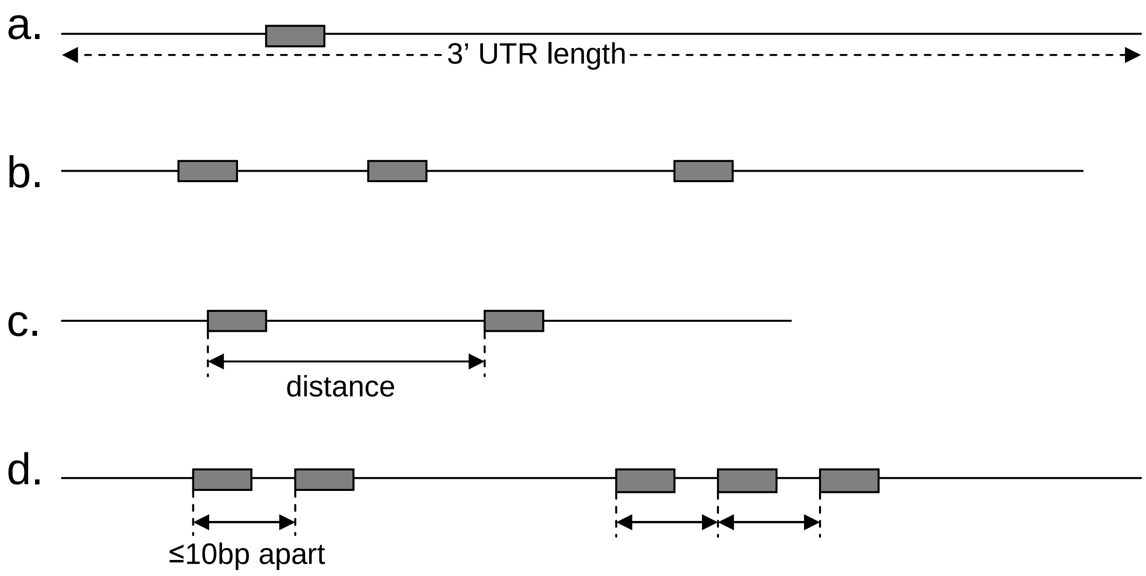

Additional Data File 1. Diagrams of experiments using RE metric. Each line represents the entire 3’ UTR length of a gene, with gray bars representing predicted target sites responsive to the same given miRNA. Other binding sites responsive to other miRNAs may also lie on these 3’ UTRs, but are not considered when calculating the RE score for this particular miRNA-mRNA interaction. (**a**) To test for the effects of 3’ UTR length on miRNA repression, miRNA-mRNA interactions containing exactly one target site responsive to the given miRNA were considered (other binding sites responsive to other miRNAs might exist on each 3’ UTR). (**b**) To test for the effects of the number of target sites on miRNA repression, miRNA-mRNA interactions involving housekeeping genes were considered. (**c**) To test for the effects of the distance between binding sites on miRNA repression, we considered each pair of binding sites responsive to the same miRNA and within 1000 bp apart, with no other binding sites responsive to the same miRNA in between. The distance between binding sites is measured from one 5’ end to the next 5’ end along the 3’ UTR, with distances of less than ~22bp constitute overlapping pairs of binding sites. (**d**) To test for the effects of having multiple pairs of extensively overlapping sites, we counted the number of pairs of sites that were within 10 bp of each other. This example contains three such pairs.
