# Supplementary material for: The roles of binding site arrangement and combinatorial targeting in microRNA repression of gene expression
Source: Genome Biol. 2007 Aug 14;8(8):R166. doi: 10.1186/gb-2007-8-8-r166 (PMC2374997; doi:10.1186/gb-2007-8-8-r166)
Supplement: Additional data file 2 — Figures using alternative data sets that support the observed trends. [file gb-2007-8-8-r166-S2.pdf]

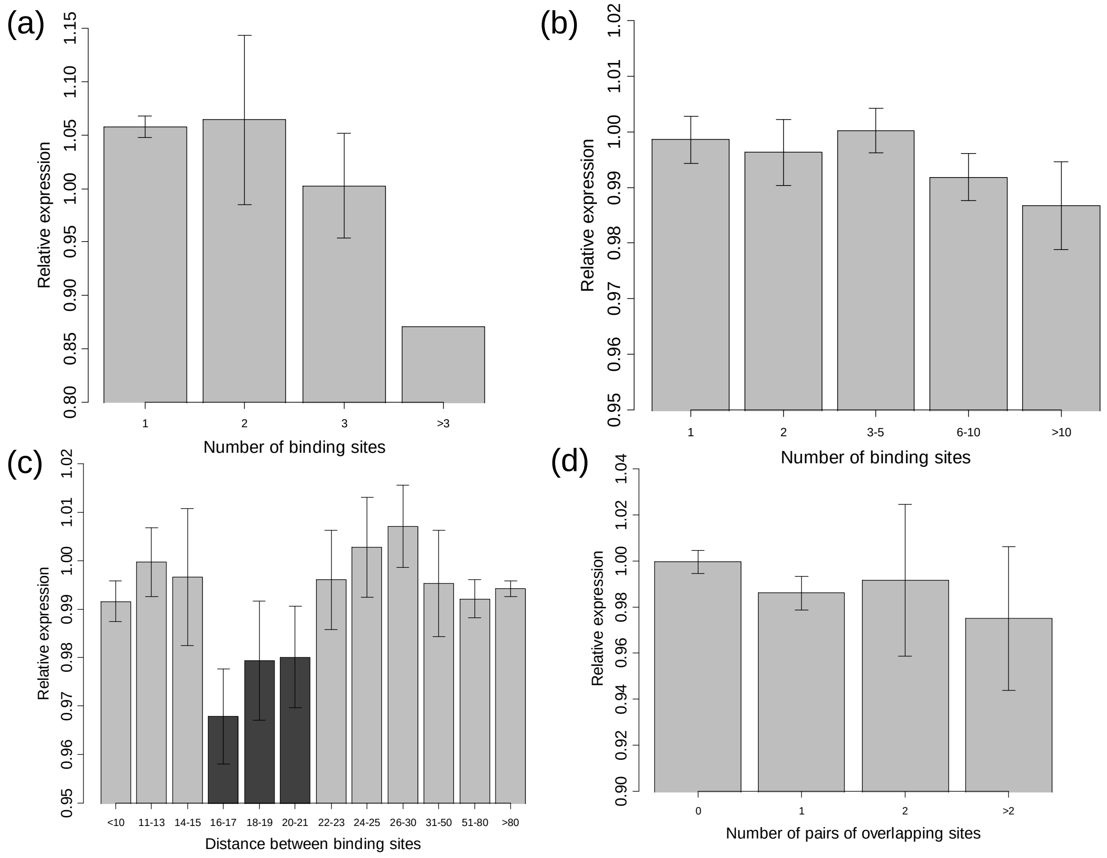

Additional Data File 2. Analysis of site and gene features that affect miRNA repression using alternative data sets. The error bars for observed data are based on the distribution of RE values. **(a)** Using target predictions from rna22, target genes containing multiple binding sites responsive to a miRNA are more strongly repressed. **(b)** Using NCI-60 expression data (and PicTar predictions), target genes with more binding sites are more strongly repressed. **(c)** Using NCI-60 data (and PicTar predictions), pairs of binding sites targeted by the same miRNA that are between 16 to 21 bp apart (by start positions) have significantly increased repression (darker gray bars shown for emphasis). **(d)** Using NCI-60 data (and PicTar predictions), genes that have multiple pairs of extensively overlapping sites, defined to be two binding sites responsive to the same miRNA whose start positions are within 10 bp of each other, have increased repression.
