# Supplementary material for: The roles of binding site arrangement and combinatorial targeting in microRNA repression of gene expression
Source: Genome Biol. 2007 Aug 14;8(8):R166. doi: 10.1186/gb-2007-8-8-r166 (PMC2374997; doi:10.1186/gb-2007-8-8-r166)
Supplement: Additional data file 3 — Description of the tables of the raw relative expression data and associated data found in Additional data files 4-8. [file gb-2007-8-8-r166-S3.pdf]

Description of Additional Data Tables

Additional Data File 4: Relative expression values for all PicTar predicted interactions using the Lu/Ramaswamy set of expression data. Columns:

- "miRNA" – name of miRNA in interaction
- "RefSeqID" – RefSeq accession of target mRNA
- "3p.UTR.length" – length of 3' UTR of target mRNA
- "Num.Binding.Sites" – number of predicted binding sites in target mRNA
- "Distances.Between.Binding.Site.Pairs" – list of distances between binding site pairs
- "Num.Pairs.of.Overlapping.Sites" – number of pairs of extensively overlapping sites
- "RE" – relative expression value
- "mir.ratio" – ratio of median miRNA expression between samples in groups A and B
- "housekeeping.gene" – is housekeeping gene? Y or N

Additional Data File 5: Relative expression values for all PicTar predicted interactions using the NCI-60 set of expression data. Columns:

- "miRNA" – name of miRNA in interaction
- "RefSeqID" – RefSeq accession of target mRNA
- "3p.UTR.length" – length of 3' UTR of target mRNA
- "Num.Binding.Sites" – number of predicted binding sites in target mRNA
- "Distances.Between.Binding.Site.Pairs" – list of distances between binding site pairs
- "Num.Pairs.of.Overlapping.Sites" – number of pairs of extensively overlapping sites
- "RE" – relative expression value
- "mir.ratio" – ratio of median miRNA expression between samples in groups A and B
- "housekeeping.gene" – is housekeeping gene? Y or N

Additional Data File 6: Relative expression values for all rna22 predicted interactions using the Lu/Ramaswamy set of expression data. Columns:

- "miRNA" – name of miRNA in interaction
- "RefSeqID" – RefSeq accession of target mRNA
- "3p.UTR.length" – length of 3' UTR of target mRNA
- "Num.Binding.Sites" – number of predicted binding sites in target mRNA
- "Distances.Between.Binding.Site.Pairs" – list of distances between binding site pairs
- "Num.Pairs.of.Overlapping.Sites" – number of pairs of extensively overlapping sites
- "RE" – relative expression value
- "mir.ratio" – ratio of median miRNA expression between samples in groups A and B
- "housekeeping.gene" – is housekeeping gene? Y or N

Additional Data File 7: Relative expression values for specific pairs of binding sites (< 1000 bp apart) responsive to a miRNA, using Lu data and PicTar predictions. Corresponds to expansion of the "Distances.Between.Binding.Site.Pairs" column in Additional Data File 4. Columns

- "miRNA" – name of miRNA in interaction
- "RefSeqID" – RefSeq accession of target mRNA
- "distance" – distance between specific binding site pair
- "utrlen" – length of the 3' UTR of the target mRNA
- "RE" – relative expression value

Additional Data File 8: Relative expression values for specific pairs of binding sites (< 1000 bp apart) responsive to a miRNA, using NCI-60 data and PicTar predictions. Corresponds to expansion of the "Distances.Between.Binding.Site.Pairs" column in Additional Data File 5. Columns

- "miRNA" – name of miRNA in interaction
- "RefSeqID" – RefSeq accession of target mRNA
- "distance" – distance between specific binding site pair
- "utrlen" – length of the 3' UTR of the target mRNA
- "RE" – relative expression value
